# Binge eating symptoms are associated with the severity of premenstrual symptoms among university students, cross sectional study from Palestine

**DOI:** 10.1186/s40337-021-00425-5

**Published:** 2021-06-09

**Authors:** Manal M. Badrasawi, Souzan J. Zidan, Nihal Natour, Israa Sharif, Shahd Atrash, Ghada Abueid, Saeda Al-Jounde

**Affiliations:** 1 Department of Nutrition and Food Technology, Faculty of Agriculture and Veterinary Medicine, An-Najah National University, PO. Box 7, Tulkarm, West Bank Palestine; 2 Department of Nutrition and Food Technology, Faculty of Agriculture, Hebron University, Hebron, West Bank Palestine; 3 Department of Public Health, Faculty of Medicine and Health Sciences, An- Najah National University, Nablus, Palestine; 4 Program of Therapeutic Nutrition, College of Medicine and Health Sciences, Palestine Polytechnic University, Hebron, Palestine

**Keywords:** Premenstrual syndrome, Binge eating disorder, Undergraduates

## Abstract

**Background:**

Premenstrual syndrome (PMS) is considered one of the most prevailing disorders among childbearing age women that could considerably interfere with daily living activities life. PMS is underrecognized in Palestine. It is reported that binge eating symptoms are significantly related to premenstrual syndrome. In this regard, the study aimed to determine factors linked with premenstrual symptoms and to explore whether binge eating symptoms are linked with premenstrual syndrome.

**Methods:**

This descriptive study was performed among female undergraduates at Palestine Polytechnic University, using a cross-sectional design. A self-administrated questionnaire was used in order to collect data. Moreover, participants’ nutritional status was assessed using anthropometric measurements. Descriptive statistics, independent t-test and Chi-square were performed.

**Results:**

A sum of 289 undergraduate females were involved in the final analysis. The results indicated that nearly half of undergraduates (47.8%) were classified as having binge eating symptoms. The most frequently noted premenstrual symptoms were lethargy, anger feelings, lack of interest, and anxiety. Chi-square analysis showed that moderate and severe psychological symptoms were significantly more prevalent among females with binge eating symptoms. Besides it was found that moderate and severe physical symptoms were significantly more prevalent among females with binge eating symptoms. Moreover, participants who reported no premenstrual syndrome symptoms were significantly less prevalent among females with binge eating symptoms. The findings also reveal that sociodemographic characteristics was not significantly correlated with premenstrual syndrome symptoms. In multiple adjusted models, both smoking (*p* < 0.05) and binge eating (*p* < 0.0001) were significantly related to PMS.

**Conclusion:**

Findings reveals that moderate and severe psychological and physical symptoms were significantly more prevalent among females with binge eating symptoms. The current research also reported that the severity of PMS was not significantly related to sociodemographic variables.

## Plain English summary

Premenstrual syndrome refers to physical, emotional, and behavioral symptoms that regularly happen in the 1 to 2 weeks before the start of each *menstrual* period. Its symptoms influence more than 90% of menstruating females. There is evidence that binge eating symptoms are significantly associated with premenstrual syndrome. The outcomes of the current study showed that half of the participated females have experienced premenstrual symptoms. There was also a significant relationship between premenstrual syndrome and binge eating symptoms.

## Introduction

Binge eating disorder (BED) is considered to be a widespread eating disorder and often occurs during late adolescence or early adulthood [1] with an estimation of a lifetime prevalence of binge eating disorder ranging from 1.9 to 2.8% [2] and previous literature showed that binge eating disorder is more common among females than males [3]. In a former Palestinian study [1], it found that the frequency of binge eating symptoms among female undergraduates was 50%.

Binge eating disorder (BED) is identified by frequent bouts of binge eating without compensative attitudes, such as diuretics or laxative use, over-training, self-induced vomiting to avoid gaining weight. The bouts of binge eating occurs along with guilty, loss of control, anxiety, and depression [4]. This disorder has the potential to decrease the quality of life, and increase the probability of developing chronic health problems. It can also increase the comorbidity with anxiety and mood disorders, and various mental issued, which may influence the magnitude of BED, and increase recovery time outcome. Premenstrual syndrome (PMS) is a possible comorbidity to BED that may impact its outset, magnitude and duration [5].

PMS is known as a group of periodic physical and emotional symptoms that manifest during the second half of the menstrual cycle (luteal phase) and stops with the outset or several days following the menses (follicular phase) [6]. The most prevalent physical and emotional symptoms involve: anger feelings, tenderness of breast, anxiety and depressed mood, abdominal bloating, and headache. Such symptoms of PMS may give rise to numerous problems, including mental health, physical impairment, and severe functional impairment in females’ occupational and social life [6].

According to the American College of Obstetrics and Gynecology (ACOG), the diagnostic criteria for PMS require complaining from at least one physical and emotional symptom during 5 days prior menses in each of three preceding menstrual cycles. Symptoms should bring about intelligible dysfunction in either economic or social achievements. This was a main point in identifying its presence and simplifying its diagnosis and its management. It is evaluated that 20–40% of childbearing age women are suffering from frequent moderate to severe PMS symptoms [7].

Findings from former studies have revealed that the prevalence of PMS symptoms is considerably great around 80–90%, and around 5% of females are having severe symptoms. Another survey also indicated that at the minimum 25% of girls aged between 14- and 15-years having PMS [8]. It is hard to evaluate the real prevalence of PMS due to a number of reasons including; definition and diagnostic criteria, self-treatment, cultural practices, variance in the availability and access to medicinal services [9]. In Palestinian frame, there is no issued data regarding PMS, as this disorder was not seen as a health issue; so, the incidence of PMS is not recognized at local level.

The etiology of PMS is unclear, multifactorial and complex and yet to be fully explained and may involve the influence of progesterone on certain neurotransmitters (e.g. serotonin, GABA, opioids, and catecholamine), elevated levels of prolactin or high sensitiveness toward the action of prolactin, insulin resistance, sensibility to endogenous hormones, altered hypothalamic-pituitary-adrenal axis role, nutritional deficiencies, changes in glucose metabolism, and alterations in fluid and electrolyte equilibrium [10].

Many thoughts result in proposing that PMS symptoms could be a risk factor for binge eating disorder. Comparable to another eating disorder, the psychological and physiological symptoms correlated with premenstrual disorder maybe a stimulus for binge eating, probably influencing the start of binge eating disorders, and it also can be as a risk factor for hindering recovery [11, 12]. Possible stimulators include physiological alterations correlated with PMS, such as body weight changes and abdominal bloating (which could be perceived as “fatness” by females with binge eating symptoms) and increases in food cravings and appetite (which could directly result in binge eating) [13].

Few studies have searched the possible role of premenstrual symptoms in the outset, duration and severity of eating disorders. Former findings have indicated that there is an acceleration of bulimic symptoms within the premenstrual period, during which elevations in the sex hormones estrogen and progesterone may be related to depression, anger, and irritability [14, 15].

Despite the mentioned verities towards premenstrual syndrome symptoms, however, there is no literature has yet assessed the prevalence of PMS and its risk factors among undergraduate females in Palestine. Thus, the most important purpose of this study is to determine the prevalence of PMS, its risk factors, and its association with binge eating symptoms among female undergraduates at Palestine Polytechnic University in Hebron city, Palestine.

## Materials and methods

### Study design, settings, and population

The current study used a cross-sectional design. The study population was undergraduate females in Palestine Polytechnic university, Hebron district- Palestine.

### Sample characteristics

The sample is drawn by random sampling, the sample size was calculated using Cochrane formula for sample size calculation in prevalence studies [16].

$$ \mathrm{n}={\left(\frac{\mathrm{z}}{\mathrm{E}}\right)}^2\mathrm{p}\left(1-\mathrm{p}\right). $$

Considering 97% confidence level (z = 2.17), estimation error (E = 0.03), and the prevalence (p = 0.5), the calculates sample size is n = 300.

The inclusion criteria were undergraduates want to take a part in the study and to provide all the needed information. While the exclusion criteria involved women who were pregnant, having cancer, undergoing hormonal therapy, having mental illness or use medication for psychiatric or psychological conditions and who rejected to take a part in the research, or turned down to confirm their participation, and those who had a missing primary data.

### Ethical consideration

The study protocol was approved by the Deanship of Scientific Research Ethical Committee at Palestine Polytechnic University committee. All undergraduate females who go to Palestinian Polytechnic university were sent a formal letter in order to participate in the study, and they were instructed about the study design, objectives, and the sort of data that would be gathered, with confirmation on the elective subscription. Undergraduate females, who concur to sign the approval letter, were involved in data gathering procedure.

### Data collection and research tool

Data collection started on January 2020 and ended on March 2020. The gathered data included sociodemographic data, self-reported medicinal history, and daily habits (smoking, and physical activity). The participants’ nutritional status was assessed using the anthropometric measures (weight and height), the weight and height measurements were done following the standard protocol by Lee and Nieman [17]. The body mass index was calculated from the weight and height thereafter classified according to WHO cut off points [18]. Physical activity level was measured by utilizing international physical activity questionnaire (IPAQ) [19]. Binge Eating Disorder Screener-7 (BEDS-7) was used to screen for binge eating symptoms [20]. PMS symptoms were assessed by utilizing the Arabic Premenstrual Syndrome Scale (APMSS) [21].

### Statistical analysis

Descriptive analysis consisting of the means (m) and the standard deviations (SD) were utilized to analyze the continuous data, whereas the categorical data were described by percentages (%) and frequencies (n). The severity of premenstrual syndrome symptoms was presented in percentages and frequencies. The relationship between premenstrual syndrome symptom and the other independent categorical variable was determined using the Chi-square test. In addition, a mixed regression model was used to identify correlates with PMS including binge eating. The model was adjusted to age, BMI, binge eating, working status, income, place of residence. All statistical analysis was performed by utilizing SPSS software version 22.

## Results

### Sociodemographic characteristics

Table 1 shows females’ distribution displayed in numbers and percentages. Two hundred eighty-nine undergraduate females took a part in the study with a mean age of 19.6 ± 1.7 years. Nearly 94 (32.5%) stated that there are in the first year, 71 (24.6%) in the second year, 52 (18.0%) in the third year, 54 (18.7) in the fourth year, while the rest 18 (6.2%) in the fifth year. About three quarters of participated females 213 (73.7%) are living in cities, whereas only 76 (26.3%) are living in either camps or villages. Most of the participants 281 (97.2%) are living with their families, while the rest 8 (2.8%) are living in university hostels. Moreover, family income for the majority 197 (68.2%) of respondents are around 1500–5000 NIS per month. About the majority of the participants 263 (91.0%) are not working, and the rest 26 (9.0%) are working either as a part-time or a full-time.

**Table 1:** Participants characteristics

Variable	Total (*N* = 289)	
	Number (N)	Percentage (%)
Faculty		
Applied science	52	18.0
Engineering	67	23.3
Medicine & health sciences	35	12.1
Applied professions	82	28.4
Administrative science & informatics	24	8.3
Information technology & computer engineering	29	10.0
Area of Living		
City	213	73.7
Village/ camp	76	26.3
Type of housing		
With family	281	97.2
University hostels	8	2.8
Family income		
< 1500 NIS	28	9.7
1500–5000 NIS	197	68.2
More than 5000 NIS	64	22.1
Working status		
Working	26	9.0
Not working	263	91.0
Study Funding		
Family	251	86.9
Scholarships	38	13.1

*NIS* Israeli New Shekel

### Lifestyle & medical history

According to Table 2, almost all the participants 281 (97.2%) stated that they are not suffering from chronic diseases, and only 6 (2.1%) of the volunteers stated that they are smokers. The findings also reveal that nearly half 167 (57.8%) of participants are engaged in mild physical activity. It was found that the participants have a mean sleeping time of 7.9 ± 1.8 h per day and have a mean screen time of 6.20 ± 3.4 h per day.

**Table 2:** Medical history & lifestyle

Variable	Total (*N* = 289)	
	Number (N)	Percentage (%)
Smoking		
Non-smoker	270	93.4
Irregular smoker	13	4.5
Regular smoker	6	2.1
Reporter type of smoking		
Cigarette	8	2.8
Pipe (shisha)	11	3.8
Chronic disease		
Yes	8	2.8
No	281	97.2
Use of medications		
Yes	43	14.9
No	246	85.1
Medical surgery		
Yes	57	19.7
No	232	80.3
Physical activity categories		
Intense	54	18.7
Moderate	68	23.5
Mild	167	57.8
	Mean	SD
Sleeping (hours/day)	7.9	1.8
Screen time (hours/day)	20	5.4

*SD* Standard deviation

### Body weight status

The findings reveal that the preponderance of the participants (72.3%) is classified with normal weight category, whereas (10.4%) were underweight, (13.5%) overweight, and (11%) obese.

### The prevalence of binge eating symptoms

Results show that nearly half of participants 138 (47.8%) were having binge eating symptoms, while the others 151 (52.2%) were not having binge eating symptoms.

### The prevalence of premenstrual syndrome symptoms

Overall, the most repeatedly noted premenstrual symptoms were lethargy (90%), anger feelings (88.2%), lack of interest (86.9%), anxiety (86.5%), depressed mood (84.7%), muscle, joint, abdominal, and back pain (84.4%), and affective liability (83.7). The most repeatedly documented severe physical symptom was muscle, joint, abdominal and back pain (32.5%). The most frequently reported moderate symptoms was anxiety (32.2%). Difficulty concentrating (38.4%), increased appetite (36.3%), craving certain food (36.0%), hypersomnia (36.0%), lethargy (34.9%), insomnia (34.9%), anxiety (34.9%), depressed mood (34.3%), headache (31.8%), hopelessness (31.8%), increased sensitivity toward others (31.8%), lack of interest (30.8%), and anger feelings (30.1%) were the most repeatedly noted mild symptoms (Table 3).

**Table 3:** Prevalence of premenstrual syndrome symptoms by the level of severity (*n* = 289)

Symptom	None	Mild	Moderate	Severe	Total^a^
	n (%)	n (%)	n (%)	n (%)	n (%)
Psychological symptoms					
Depressed mood	44 (15.2)	99 (34.3)	85 (29.4)	61 (21.1)	245 (84.7)
Hopelessness	80 (27.7)	92 (31.8)	75 (26.0)	42 (14.5)	209 (72.3)
Guilt feeling	125 (43.3)	86 (29.8)	47 (16.3)	31 (10.7)	164 (56.7)
Anxiety/ worry	39 (13.5)	101 (34.9)	93 (32.2)	56 (19.4)	250 (86.5)
Affective labiality	47 (16.3)	77 (26.6)	87 (30.1)	78 (27.0)	242 (83.7)
Increased sensitivity toward others	62 (21.5)	92 (31.8)	70 (24.2)	65 (22.5)	227 (78.5)
Anger feelings	34 (11.8)	87 (30.1)	92 (31.8)	76 (26.3)	255 (88.2)
Easily irritated/ agitated	59 (20.4)	74 (25.6)	86 (29.8)	70 (24.2)	230 (79.6)
Lack of interest	38 (13.1)	89 (30.8)	92 (31.8)	70 (24.2)	251 (86.9)
Difficulty concentrating	60 (20.8)	111 (38.4)	66 (22.8)	52 (18.0)	229 (79.2)
Loss of control	75 (26.0)	86 (29.8)	62 (21.5)	66 (22.8)	214 (74.0)
Feeling overwhelmed	69 (23.9)	84 (29.1)	76 (26.3)	60 (20.8)	220 (76.1)
Physical symptoms					
Lethargy/ fatigue/ decreased energy	29 (10.0)	101 (34.9)	75 (26.0)	84 (29.1)	260 (90.0)
Increased appetite	83 (28.7)	105 (36.3)	53 (18.3)	48 (16.6)	206 (71.3)
Craving certain foods	81 (28.0)	104 (36.0)	51 (17.6)	53 (18.3)	208 (72.0)
Hypersomnia	58 (20.1)	104 (36.0)	73 (25.3)	54 (18.7)	231 (79.9)
Insomnia	71 (24.6)	101 (34.9)	69 (23.9)	48 (16.6)	218 (75.4)
Breast tenderness	113 (39.1)	84 (29.1)	39 (13.5)	53 (18.3)	176 (60.9)
Breast engorgement or weight gain	109 (37.7)	83 (28.7)	45 (15.6)	52 (18.0)	180 (62.3)
Headache	61 (21.1)	92 (31.8)	71 (24.6)	65 (22.5)	228 (78.9)
Muscle, joint, abdominal, and back pain	45 (15.6)	82 (28.4)	68 (23.5)	94 (32.5)	244 (84.4)
Acne	69 (23.9)	81 (28.0)	71 (24.6)	68 (23.5)	220 (76.1)
Behavioural symptoms					
Symptoms interfering with					
Relationships	104 (36.0)	82 (28.4)	59 (20.4)	44 (15.2)	185 (64.0)
Work or school	114 (39.4)	84 (29.1)	60 (20.8)	31 (10.7)	175 (60.6)
Overall psychological symptoms	33 (11.4)	107 (37.0)	101 (34.9)	48 (16.6)	256 (88.6)
Overall physical symptoms	28 (9.7)	128 (44.3)	88 (30.4)	45 (15.6)	261 (90.3)
Overall assessment of behavioural symptoms	85 (29.4)	89 (30.8)	81 (28.0)	34 (11.8)	204 (70.6)

^a^Sum of mild, moderate, and severe symptoms only

### Relationship between premenstrual syndrome symptoms & binge eating symptoms

Findings reveal that moderate (43.5%) and severe (23.2%) psychological symptoms were significantly more prevalent among females with binge eating symptoms (*p* < 0.05). Besides it was found that moderate (35.5%) and severe (22.5%) physical symptoms were significantly more prevalent among females with binge eating symptoms (*p* < 0.05). The analysis also reveals that moderate behavioral symptoms (42.8%) were significantly more prevalent among females with binge eating symptoms (*p* < 0.05). Moreover, participants who reported no premenstrual syndrome symptoms were significantly less prevalent among females with binge eating symptoms (*p* < 0.05) as demonstrated in Table 4.

**Table 4:** Relationship between premenstrual syndrome symptoms & binge eating symptoms

	Females with binge eating symptoms (*n* = 138)	Females without binge eating symptoms (*n* = 151)	*P*-value
Psychological symptoms			
None	7 (5.1)	26 (17.2)	<0.01^*^
Mild	39 (28.3)	68 (45.0)	
Moderate	60 (43.5)	41 (27.2)	
Severe	32 (23.2)	16 (10.6)	
Physical symptoms			
None	6 (4.3)	22 (14.6)	<0.01*
Mild	52 (37.7)	76 (50.3)	
Moderate	49 (35.5)	39 (25.8)	
Severe	31 (22.5)	14 (9.3)	
Behavioural symptoms			
None	24 (17.4)	61 (40.4)	<0.01^*^
Mild	39 (28.3)	50 (33.1)	
Moderate	59 (42.8)	22 (14.6)	
Severe	16 (11.6)	18 (11.9)	

*significant at *p*< 0.01 using chi sqaure test

### Relationship between premenstrual syndrome symptoms & sociodemographic characteristics

Our results reveal that premenstrual syndrome symptoms were not significantly related to sociodemographic characteristics (working status, family income, area of living, type of housing, and study expenses).

Finally, using multiple logistic regression models to study the relationship between binge eating and PMS, higher PMS score was associated with increased odds of binge eating in models adjusted for age, BMI, place of residence, smoking, work and income. Being from city almost doubled the risk of have binge eating. Also, not smoking or irregular smoking was associated with increased odds of binge eating, whereas work and education were not significantly related odds of having binge eating, we included in the adjustment variables we believed that they define our participants and could have impact on eating habits as is found (Table 5).

**Table 5:** Multiple logistic regression association between binge eating symptoms and PMS score

Variable	Beta	Wald	***P***-value	OR	Lower CI	Upper CI
Age (y)	−0.03	0.10	0.70	1.03	0.88	1.2
BMI (Kg/m^2^)	−0.06	2.12	0.15	1.06	0.98	1.16
PMS Score	−0.05	29.16	< 0.0001	1.05	1.03	1.06
**Place:** City versus Village	−0.67	4.95	0.03	1.96	1.09	3.45
**Smoking**						
Smoking (reference)						
Non-smoker	−2.78	4.09	0.04	16.67	1.09	250
Irregular smoker	−2.56	2.96	0.09	12.5	0.70	250
**Work**						
Not working (reference)						
Full time	1.78	1.67	0.20	0.17	0.01	2.5
Part time	0.06	0.01	0.93	0.94	0.26	3.45
**Income**	−0.001	2.12	0.15	1.00	1.00	1.01

## Discussion

The current study was performed originally to define the prevalence and severity of PMS symptoms among a sample of female undergraduates, to determine related sociodemographic factors, and to verify whether premenstrual syndrome symptoms is correlated with binge eating symptoms. Based on the current literature, this is the first research that explored the prevalence of PMS among female undergraduates in Palestine, and the first one that examined its association with binge eating symptoms.

Similar to former studies [22–24], we have noticed that anger/ irritability was the most repeatedly reported psychological symptom. Moreover, abdominal pain is one of the most commonly documented physical symptoms among participants in the present study. This finding is in line with former studies [25–28]. However, this finding inconsistent with former studies conducted in China [29] and in India [30]. This difference in the nature of documented physical symptoms could be elucidated by the fact that female undergraduates follow different lifestyle and dietary practices prior and during their menstruation.

Furthermore, our analysis reveals that the least frequent severe symptoms were behavioral symptoms by 11.8% (especially, PMS symptoms influencing school or work). This finding supports a former study that found behavioral symptoms was the least common symptom by 2.0% [25].

In the current study, PMS symptoms were not significantly associated with residence nor family income. In a former study performed by Marván and his collogues, however, it was noted that lower socioeconomic status is associated with higher incidence of PMS [31]. In a recent study, also, it was showed that higher income household and urban residence were significantly related with the risk of PMS. Additional studies are needed to find out the exact association between socioeconomic status and the prevalence of PMS symptoms.

It has been suggested that cigarette smoking result in an imbalance in estrogen, progesterone, androgen, and gonadotropin concentrations, which may be a cause of PMS [32]. Our results were consistent with former findings where it is found that smoking is associated with increased premenstrual symptoms [33, 34].

In the current study, physical activity level was not significantly related to PMS symptoms. This was confirmed by previous studies conducted in United Arab Emirates [25] and United Kingdom [35], where it was showed that physical exercise is not correlated to PMS symptoms. Conversely, Teixeira and colleagues reported that there is a negative relationship between the level of physical activity and the prevalence of PMS [36]. Further research in this context is needed.

Besides this study found that moderate and severe psychological and physiological symptoms were significantly more prevalent among females with binge eating symptoms. Former studies indicated that it is probable that females with premenstrual dysphoric disorder (PMDD) are at high risk for giving rise to binge eating disorder because they bear increased premenstrual negative influence and appetite for calorie-dense foods, which, in turn, results in binge eating [37, 38]. It is also probable that some females are, in particular, sensible to the effect of ovarian hormones, and that differences in ovarian hormones may elevate the risk for both binge eating and negative impact, thus elevating the risk of binge eating disorder. Future studies are necessary to verify if the outset of premenstrual conditions forgo the start of binge eating, and subsequently may give rise to the development of binge eating disorder [5].

There were several limitations in the current study. Firstly; the major limitation of the current study resides in its design. Being cross-sectional, it’s impossible to derive a cause-effect relationship. Secondly; the study was limited to Palestine Polytechnic University in Hebron city in Palestine and does not exemplify the whole female undergraduates’ category in Palestine. Thirdly; since this object is ticklish for the Palestinian community, some participants might not desire to uncover their actual personal issues. Nevertheless, the present research gives for valuable information toward the relationship between PMS and binge eating symptoms. Additional studies should be pointed from the overall results regarding investigating the hormonal, molecular and genetic alterations correlated with PMS among Palestinian female undergraduates. Moreover, future studies should focus on clarifying the causal relationship for better understanding of PMS.

## Conclusion

This study is considered one of its kind examining PMS among female undergraduates in Palestine and pointing out the high prevalence of PMS among this category. Interestingly, the findings reported that moderate and severe psychological symptoms were significantly more prevalent among females with binge eating symptoms. Besides it was found that moderate and severe physical symptoms were significantly more prevalent among females with binge eating symptoms. The current study documented that PMS is associated with smoking. There is a necessity for better diagnosis of PMS among female undergraduates so that they will not feel reluctant to search for a appropriate medicinal counsel.

## Data Availability

The dataset used and analysed in this study is available from corresponding Author on reasonable request.

## Abbreviations

BED: Binge eating disorder
PMS: Premenstrual Syndrome
PMDD: Premenstrual Dysphoric Disorder
